# FOLLOWING IN THE FOOTSTEPS OF A GREAT APPLIED GERONTOLOGIST (WHILE SIMULTANEOUSLY FORGING YOUR OWN PATH)

**DOI:** 10.1093/geroni/igz038.1599

**Published:** 2019-11-08

**Authors:** Carol Whitlatch

**Affiliations:** Benjamin Rose Institute on Aging, Cleveland, Ohio, United States

## Abstract

Dr. M. Powell Lawton was an inspirational and productive scholar whose work had application for improving personal care and well-being for older adults and their families. He held strong to the principles of Person- and Family-Centered Care long before this terminology and model of care was commonly practiced. He was a living example of PFCC always wearing comfortable clothes and shoes to ensure his unique creativity was not obstructed by physical discomfort. Dr. Lawton’s work has inspired countless gerontologists who have taken the next steps towards ensuring quality of care by understanding personal preferences, activities, and care values. Dr. Whitlatch will discuss Dr. Lawton’s ongoing influence on care and best practices focusing on her own research that gives voice to the care values, preferences, and activities of families facing early-stage dementia. Dr. Whitlatch encourages attendees to wear their most comfortable shoes to the lecture in honor of Dr. Lawton.

